# Dietary Fructose Intake and Hippocampal Structure and Connectivity during Childhood

**DOI:** 10.3390/nu12040909

**Published:** 2020-03-26

**Authors:** Kristi A. Clark, Jasmin M. Alves, Sabrina Jones, Alexandra G. Yunker, Shan Luo, Ryan P. Cabeen, Brendan Angelo, Anny H. Xiang, Kathleen A. Page

**Affiliations:** 1 Department of Neurology, Keck School of Medicine of the University of Southern California, Los Angeles, CA 90033, USA; kristi.clark.999@gmail.com; 2 Department of Internal Medicine; Division of Endocrinology, Keck School of Medicine of the University of Southern California, Los Angeles, CA 90033, USA; jalves@usc.edu (J.M.A.); sljones@usc.edu (S.J.); ayunker@usc.edu (A.G.Y.); shanluo@usc.edu (S.L.); angelob@usc.edu (B.A.); 3 Diabetes and Obesity Research Institute of the University of Southern California, Los Angeles, CA 90033, USA; 4 Department of Psychology, University of Southern California, Los Angeles, CA 90089, USA; 5 Laboratory of Neuro Imaging (LONI), USC Stevens Neuroimaging and Informatics Institute, Keck School of Medicine of USC, Los Angeles, CA 90033, USA; Ryan.Cabeen@loni.usc.edu; 6 Department of Research and Evaluation, Kaiser Permanente Southern California, Pasadena, CA 91101, USA; Anny.H.Xiang@kp.org

**Keywords:** children, cingulum, development, dietary sugar, fructose, hippocampus

## Abstract

In rodent literature, there is evidence that excessive fructose consumption during development has a detrimental impact on hippocampal structure and function. In this study of 103 children ages 7–11 years old, we investigated whether dietary fructose intake was related to alterations in hippocampal volume and connectivity in humans. To examine if these associations were specific to fructose or were related to dietary sugars intake in general, we explored relationships between dietary intake of added sugars and the monosaccharide, glucose, on the same brain measures. We found that increased dietary intake of fructose, measured as a percentage of total calories, was associated with both an increase in the volume of the CA2/3 subfield of the right hippocampus and increased axial, radial, and mean diffusivity in the prefrontal connections of the right cingulum. These findings are consistent with the idea that increased fructose consumption during childhood may be associated with an inflammatory process, and/or decreases or delays in myelination and/or pruning. Increased habitual consumption of glucose or added sugar in general were associated with an increased volume of right CA2/3, but not with any changes in the connectivity of the hippocampus. These findings support animal data suggesting that higher dietary intake of added sugars, particularly fructose, are associated with alterations in hippocampal structure and connectivity during childhood.

## 1. Introduction

Diets high in added sugars, particularly fructose, have adverse metabolic consequences and are associated with oxidative stress, insulin resistance, and cardiometabolic disorders [1,2,3,4]. Beyond its known metabolic health risks, high fructose consumption has been linked to impairments in peripheral and central appetite signaling and may promote feeding behavior [5,6,7,8,9,10]. Emerging data also indicates that high fructose diets have a profound impact on brain function [11,12], particularly within the hippocampus, a brain region involved in memory, learning, and food intake regulation that is particularly vulnerable to dietary and metabolic insults [13,14,15]. Experimental studies in animal models have shown that high fructose consumption leads to hippocampal insulin resistance [16,17], neuroinflammation [18,19], and reduced hippocampal neurogenesis [20], and suggests a potential mechanistic basis for fructose induced cognitive deficits [17,18,21].

The findings that hippocampal function is highly sensitive to excess fructose consumption are particularly relevant to the study of obesity and excess weight gain considering the role the hippocampus plays in energy regulation. In rodents, hippocampal lesions [14] or inactivation [15] increase food consumption and body weight. The translation of these findings to humans has been supported by experiments in patients with brain lesions. Patients with lesions to the temporal lobe have been shown to overeat in response to food presentation relative to healthy controls and patients with lesions in other brain regions, even after consuming an energy dense meal [22,23]. Considering these findings together, the hippocampus appears to be highly important in the inhibition of food consumption; therefore, in the case of hippocampal impairment by external insults, such as excessive consumption of fructose, humans and animals may overeat and consequently gain additional weight.

Human hippocampal connectivity has been found to be quite extensive [24], and evidence from animal models suggests that the ability of the hippocampus to inhibit appetitive behaviors may rely on its connections to other brain areas. Prior findings have demonstrated that disconnection lesions (the elimination of all direct and indirect connections) between the ventral hippocampus and the contralateral prefrontal cortex in rodents impairs general behavioral inhibition in a 5-choice reaction time task [25]. More specifically related to obesity, a monosynaptic glutamatergic pathway between the ventral hippocampus and the medial prefrontal cortex has been identified as important in the control of energy regulation. Chemogenic inactivation of this pathway has been shown to increase food consumption, indicating the activation of this pathway may be important in the ability to suppress overeating [26]. The hippocampus is also connected to the lateral septum, and activation of this excitatory pathway inhibits food intake [27]. Other hippocampal-dependent feeding pathways include connections with the amygdala and hypothalamus [18,28]. While diets high in fructose or other added sugars have been found to alter hippocampal physiology [18,20], and metabolic insults, such as obesity, have been shown to decrease hippocampal global brain connectivity [29], the impact of dietary fructose or added sugar intake on the microstructure of pathways that connect the hippocampus to other brain regions has not yet been studied.

Recent studies in rodents have shown that the effects of high fructose diets on hippocampal function are particularly damaging during sensitive periods of neurocognitive development, such as childhood and adolescence [18], but few studies have attempted to translate these findings to humans. To address this gap in knowledge, we used in vivo magnetic resonance imaging (MRI) methods, which provide a non-invasive way to examine the neurodevelopment of the human brain. T1-weighted acquisitions allow quantification of grey matter volume in specific regions of the brain, and diffusion tensor imaging (DTI) is a sensitive imaging method that can characterize the microstructural architecture of white matter tracts that connect distinct brain regions [30]. We used both structural MRI and diffusion MRI to examine the associations between dietary fructose and added sugar intake and hippocampal development in healthy children aged 7 to 11 years. Based on studies in animal models, we hypothesized that higher dietary intake of added sugars, particularly fructose, would be associated with alterations in hippocampal volume and in the microstructure of white matter tracts that connect the hippocampus to other brain regions.

## 2. Materials and Methods

### 2.1. Participants

Healthy, typically developing children between the ages of 7–11 years old participated in the BrainChild Study of the impact of intrauterine exposure to metabolic disorders on brain pathways during childhood. Children were born at Kaiser Permanente Southern California (KPSC), a large health care organization that uses an integrated electronic medical record (EMR) system. KPSC member demographics are broadly representative of Southern California residents [31]. Each participating Institutional Review Board approved this study (University of Southern California (USC) #HS-14-00034, KPSC #10282). Participants’ parents gave written informed consent. Children provided written informed assent.

The study included two in-person visits. Visit one occurred at the Clinical Research Unit of the USC Diabetes and Obesity Research Institute. Visit two occurred at USC Dana and David Dornsife Neuroimaging Center and included a MRI scan of the brain.

### 2.2. Clinical Characteristics and Demographics

Child’s height was measured to the nearest 0.1 cm using a stadiometer and weight to the nearest 0.1 kg using a calibrated digital scale. BMI was calculated using the standard formula, weight (kg) divided by height (m^2^). BMI z-scores and BMI percentiles (age and sex-specific standard deviation scores) were determined based on Center for Disease Control (CDC) standards [32]. Participants were given the option of having Tanner stage assessed by physical exam [33,34] and/or by a validated sex-specific assessment questionnaire for children and parents, containing both illustrations and explanatory text [35]. Forty-eight participants opted for both physical exam and questionnaire, and 55 participants opted for self-reported puberty status only. The correlation between Tanner Staging assessed by physical exam and by questionnaire was 0.91.

Socioeconomic status (SES) was assessed using household income at birth, estimated based on census tract of residence and expressed as a continuous variable, and maternal education at birth, which was extracted from birth certificates in the EMR as a categorical variable with the following categories: “high-school or some high-school”, “some college”, and “college and post-education”. Prenatal exposures to maternal gestational diabetes mellitus (GDM) and maternal pre-pregnancy BMI were assessed using the EMR.

### 2.3. Dietary Measures

Diet was assessed using repeated 24-h dietary recalls on non-consecutive days during two in-person visits as part of the BrainChild Study. In a subset of 35 participants, we also obtained 3-day dietary records on one of the two in-person visits, and dietary intake was estimated using a total of four days of dietary assessments for these participants. The mean interval between the two in-person visits was 34 ± 57 days. We used the multi-pass method for dietary recall, in which a trained staff member asked the participant to recall what food and beverages they had consumed over a 24-h time period with the input of both the child and the child’s parent. The trained staff member then went through three other “passes” to complete quantity of food/beverages consumed as well as to include missing or forgotten food/beverages. Use of the multi-pass 24-h dietary recall method is a valid method to assess energy intake in children [36]. Once the dietary recalls were collected, the recalls were analyzed using the Nutritional Data System for Research software v.2018 developed by the Nutrition Coordinating Center (NCC), University of Minnesota, Minneapolis, MS [37]. The variables used were percent calories from fructose, percent calories from glucose, and percent calories from added sugar by available carbohydrate. Data from dietary recalls were manually checked for quality. To determine outliers, we performed linear regression analysis, using body weight to predict total energy intake. Residuals were standardized and examined for any values that were >3 or <−3 standard deviations from the mean. Records containing data that exceeded these values were not included in the analysis. Using this method, 267 dietary recalls were included in the analysis, and one dietary recall was excluded.

### 2.4. Magnetic Resonance Imaging (MRI) Acquisition

After a mock scanner training session, magnetic resonance imaging (MRI) was performed using a Siemens MAGNETOM Prisma^fit^ 3 Tesla MRI scanner (Siemens Medical Systems) with a 20-channel phased array coil. A high-resolution magnetic resonance imaging scan was acquired using a T1-weighted three-dimensional magnetization prepared rapid gradient echo (MP-RAGE) sequence with the parameters: 256 × 256 × 176-matrix size with 1 × 1 × 1-mm^3^ resolution; inversion time = 900 ms; repetition time (TR) = 1950 ms; echo time (TE) = 2.26 ms; flip angle = 90°; Total scan duration was 4 min and 14 s. A diffusion weighted image was acquired using a dual spin echo, single shot, pulsed gradient, echo planar imaging sequence in 64 diffusion sensitized gradient directions with the following parameters: TR = 8100 ms; TE = 69 ms; flip angle = 90°; 70 axial slices; 2 mm × 2 mm × 2 mm voxel size; FOV = 256 mm; *b* value = 1000 s/mm^2^; Total scan duration was 9 min and 29 s.

### 2.5. MRI Data Processing

The T1 MP-RAGE structural image was put into the automated segmentation software, FreeSurfer version 6.0 hippocampal module (http://surfer.nmr.mgh.harvard.edu/, RRID:SCR_001847) to examine total hippocampal grey matter volume and grey matter volume in the hippocampal subfields. The procedure uses Bayesian inference and a probabilistic atlas of the hippocampal formation based on manual delineations of subfields in ultra-high-resolution MRI scans [38]. Manual quality check of automated hippocampal segmentation was performed for each participant following an existing protocol [39]. The segmentation of the hippocampus was visually assessed by an individual trained in hippocampal neuroanatomy and then given a rating of “pass”, “pass on condition”, and “fail”. Images that failed to have defined landmarks due to motion artifacts or segmentation error were excluded. Although twelve subfield volumes are generated by FreeSurfer 6.0, we only included subfields that have been shown to be preferentially affected by high sugar diets, including the CA1, CA2/3, CA4, DG (granule cell layer) and subiculum [40,41]. Previous studies in children have used FreeSurfer to segment the hippocampus and hippocampal subfields [42,43]. The raw volume data were included in the supplemental materials (Table S1).

**Figure 1:**
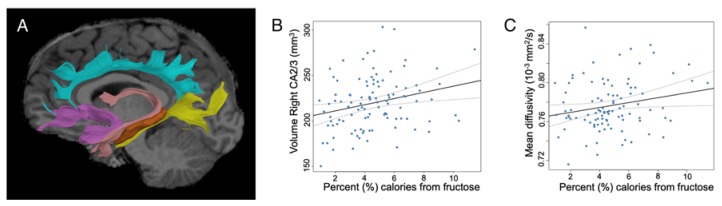
Associations with fructose consumption. (**A**) Using regions of interest for the hippocampus (orange) and its major connections (fornix: pink, uncinate: purple, and the cingulum, which was segmented into the temporal part: yellow and the prefrontal part: turquoise), we identified associations between fructose consumption and the volume of the right CA2/3 subfield of the hippocampus (**B**) and the mean diffusivity of the right cingulum, prefrontal connections (**C**). (**B**,**C**) Graphs show the unadjusted model with no covariates. Solid line indicates the best fit linear trend, with dotted lines showing the confidence interval. Notably, these graphs show the results from Model 1 (Table 1 and Table 2, respectively).

Tractography models were created from the diffusion-weighted MRI (dMRI) data using FSL [44] and the Quantitative Imaging Toolkit (QIT) [45]. The dMRIs were first skull stripped using FSL BET and then corrected for motion and eddy current artifact using FSL FLIRT. For this, each diffusion scan was affinely registered to the baseline scan using the mutual information metric, and the associated gradient orientations were rotated to account for the registration. Diffusion tensor models were then estimated from the dMRI using QIT, and the following tensor parameters were extracted: fractional anisotropy (FA), mean diffusivity (MD), axial diffusivity (AD), and radial diffusivity (RD). Tensor images were upsampled to 1 mm^3^ using model-based interpolation in QIT [46], and a deformation field was computed using DTI-TK [47] to register the data to the IIT brain template [48]. Tractography models of the bundles-of-interest were created using a framework for deterministic streamline integration [49]. For each bundle, seed, inclusion and exclusion masks were manually drawn in the IIT template [50] in reference to a white matter atlas [51]. The template masks were then resampled in each subject’s native space image to constrain tractography. Other tractography parameters included a step size of 1.0 mm, a maximum angle of 45 degrees, a minimum FA of 0.15, and 25000 seeds per bundle. Bundle-specific metrics of fractional anisotropy (FA), mean diffusivity (MD), axial diffusivity (AD), and radial diffusivity (RD) were then computed. FA and MD were used as primary metrics of microstructure, with many studies showing that FA increases with age while MD decreases with age [52,53,54,55]. AD and RD were used as post-hoc measures in regions that showed a significant effect on MD. The MD measure is a weighted average of AD and RD, which themselves are more biologically specific than MD alone. The bundles of interest included major connections between the hippocampus and the rest of the brain, specifically: the uncinate fasciculus, fornix, and cingulum bundle (separated into prefrontal and temporal lobe sections) (See Figure 1A).

### 2.6. Statistical Analysis

Hierarchical regression models were implemented in R to estimate the effects of dietary consumption of fructose on the volume of the hippocampus and its connectivity with the rest of the brain. The dependent variables for the volume of the hippocampus included the following regions of interest (ROIs): (i) Whole hippocampus, (ii) CA1, (iii) CA2/3, (iv) CA4, (v) DG/GC/ML, (vi) subiculum. Left and right values were tested separately for a total of 12 tests, which led to a per-test p-value of 0.004 to reach an overall alpha of *p* < 0.05 using Bonferroni correction. The dependent variables for the connectivity between the hippocampus and the rest of the brain included the following tracts: (i) uncinate, (ii) fornix, (iii) cingulum–prefrontal section, and (iv) cingulum–temporal section. Left and right values were tested separately and MD and FA were tested separately for a total of 16 tests, which led to a per-test p-value of 0.003 to reach an overall alpha of *p* < 0.05 using Bonferroni correction. Planned post-hocs included: testing the associations of dietary added sugar and glucose intake on brain metrics that showed evidence of being affected by fructose, and testing the effects of axial and radial diffusivity for any tests that showed significant effects in FA or MD. Associations of sugar consumption with total intracranial volume were also computed as a negative control.

Model 1 was the unadjusted model for percent calories from fructose. Model 2 included age and sex (and intracranial volume for analyses of the volume of the hippocampus). Model 3 included the child’s BMI z-score. Model 4 included two categorical variables aimed at measuring socioeconomic status: the highest education level attained by the mother and the family income level. Model 5 included maternal measures that impact prenatal environment, i.e., gestational diabetes (binary) and pre-pregnancy BMI. As variables were added, an F-test was used to determine whether the newly added variables showed a significant improvement in R^2^. Semi-partial r values were calculated as an indication of effect size, where r < 0.1 is a small effect size, 0.1 < r < 0.3 is a medium effect size, and r > 0.3 is a large effect size.

Effects of Tanner stage and potential interactions with sex were tested as Models 2A and 2B respectively. If either or both were found to explain significantly more variance than Model 2, then models 3–5 included a covariate for Tanner stage and/or an interaction between the dietary measure and sex.

## 3. Results

### 3.1. Participant Characteristics

A total of 103 children participated in this study. Child demographic and clinical characteristics are reported in Table 1. The mean ± SD age of the children was 8.55 ± 1.03 years old, 91% of the children were pre-pubertal (Tanner Stage <2), and 62% were girls. Children’s BMI ranged from 13.62 to 34.01 kg/m^2^; BMI percentiles ranged from 5.28 to 99.58; BMI z-scores ranged from −1.78 to 2.64. According to CDC standards, 61 (59%) children were healthy-weight, 16 (15%) children were overweight, and 26 (25%) children were obese. Overall, the participants consumed an average (±SD) of 1763 ± 359 kcal per day; 4.57 ± 2.19% of their total calories came from fructose, 4.32 ± 1.81% of their total calories came from glucose, and 13.91 ± 6.89% of total calories from added sugar. Total energy and added sugar intake in our cohort are in line with the general population of the children in the United States [56]. Familial demographics, including maternal education, family income, and mother’s self-reported race/ethnicity, are shown in Table 2. Maternal pre-pregnancy BMI ranged from 18.97 to 50.38 kg/m^2^, and 58% of mothers had GDM during pregnancy.

### 3.2. Influence of Diet on Hippocampal Volumes

A total of 101 participants were included in the hippocampal volume analyses, after excluding the two participants who were missing data for the mother’s education level. The amount of fructose children consumed as a percentage of calories in their diet was significantly associated with an increase in the volume of the right CA2/3 hippocampal subfield (ß = 3.34, sr = 0.25, *p* < 0.01; Model 1)) (Table 3; Figure 1B). Results remained significant after adjusting for ICV, age, and sex (ß = 2.56, sr = 0.19, *p* < 0.02; Model 2), further adjusting for child BMI z-score (ß = 2.80, sr = 0.21, *p* < 0.01; Model 3), and additionally adjusting for SES (ß = 2.70, sr = 0.19, *p* < 0.02; Model 4), as well as in fully adjusted models (ß = 3.33, sr = 0.24, *p* < 0.003; Model 5). These findings suggest that dietary fructose has an effect on hippocampal CA 2/3 volume that is independent of child’s age, sex, BMI Z-score, SES, or prenatal exposures. Of note, Model 2, which included covariates for intracranial volume, age, and sex, explained significantly more variance than the unadjusted Model 1, which only contained a covariate for percentage of calories from fructose (F(3,96) = 16.78, *p* < 0.001). This result is unsurprising because the volume of the hippocampus and its subfields are highly dependent upon total brain volume. Model 5, which included all covariates, explained significantly more variance than Model 4, which included all covariates except maternal GDM status and maternal pre-pregnancy BMI (F(2,90) = 6.56, *p* < 0.002). All other hippocampal subfield volumes were non-significant. None of the models tested showed significant interactions of sex with dietary intake, nor did they show significant effects of Tanner stage. Additionally, neither fructose (ß = 6893, *p* < 0.30, R^2^ = 0.0008), glucose (ß = 7583, *p* < 0.30, R^2^ = 0.0008), nor added sugar consumption (ß = −277, *p* < 0.89, R^2^ = −0.0097) were significantly associated with intracranial volume.

In planned post-hoc analyses, we explored whether the associations between dietary fructose intake and hippocampal CA 2/3 volume were specific to fructose, or if alternatively the associations were also observed between dietary added sugar and/or glucose intake and CA 2/3 volume. We found that associations between percent of calories from added sugar and volume of the right CA2/3 were also significant (R^2^ = 0.448, F(10,90) = 7.29, *p* < 0.001). Likewise, planned post-hoc analyses on the effects of percent of calories from glucose on the volume of the right CA2/3 were also significant (R^2^ = 0.471, F(10,90) = 8.00, *p* < 0.001). These findings indicated that effects of dietary fructose, added sugar, and glucose intake on right CA2/3 volume were all similar suggesting that increases in the volume of right CA2/3 were non-specific with regards to whether the increased sugar intake was from glucose, fructose, or added sugars. In fact, the effect sizes for glucose intake (sr = 0.27), fructose intake (sr = 0.25), and added sugar intake (sr = 0.23) were nearly identical, indicating each of these types of sugar explained a similar amount of variance. The percent calories from fructose and percent calories from glucose were highly correlated (r = 0.87, *p* < 0.001), and there were moderate correlations between percent calories from fructose and percent calories from added sugar (r = 0.31, *p* < 0.001) and between percent calories from glucose and percent calories from added sugar (r = 0.48, *p* < 0.001). Given that dietary intake of fructose, glucose, and added sugar were correlated, we did not include them in the same model to test independent effects. It is important to note that added sugars are classified as sugars that are added to foods or beverages when they are processed or prepared, whereas naturally occurring sugars, such as those in fruit or milk, are not classified as added sugars [57].

### 3.3. Influence of Diet on Hippocampal Connectivity

A total of 98 participants were included in the diffusion imaging analyses, after excluding the 6 participants who were missing data for either the mother’s education level (*n* = 2) or who failed quality control for the diffusion imaging data (*n* = 4). The amount of fructose children consumed as a percentage of calories was significantly associated with an increase in the mean diffusivity (MD) of the right cingulum, prefrontal connections (ß = 2.51 × 10^−6^, sr = 0.21, *p* < 0.04; Model 1) (Table 4; Figure 1C). Results remained significant after adjusting for age and sex (ß = 2.66 × 10^−6^, sr = 0.22, *p* < 0.02; Model 2), further adjusting for child BMI z-score (ß = 2.79 × 10^−6^, sr = 0.23, *p* < 0.02; Model 3), and additionally adjusting for SES (ß = 3.51 × 10^−6^, sr = 0.28, *p* < 0.003; Model 4), as well as in fully adjusted models (ß = 3.64 × 10^−6^, sr = 0.29, *p* < 0.002; Model 5). The outcome of the hierarchical modeling indicated that Model 4 explained significantly more variance than Models 1–3, but Model 5 did not explain more variance than Model 4. Therefore, Model 4 was used for the planned post-hoc analyses. The MD for all other tracts and the FA for all the tracts were non-significant. None of the models tested showed significant interactions of sex with dietary intake, nor did they show significant effects of Tanner stage.

Planned post-hoc analyses on the effects of percent of calories from added sugar on the MD of the right cingulum, prefrontal connections resulted in a significant model overall (R^2^ = 0.167, F(7,90) = 2.58, *p* < 0.02). Although the overall model was significant, the covariate of interest (i.e., percent calories from added sugar) was not significantly associated with MD of the right cingulum, prefrontal connections (ß = −2.56 × 10^−7^, sr = −0.07, *p* < 0.50), indicating that the overall model for added sugar associations with MD of the right cingulum, prefrontal connections was driven by significant associations with decreased age and increased family income. Planned post-hoc analyses on the effects of percent of calories from glucose on the MD of the right cingulum, prefrontal connections also resulted in a significant model overall (R^2^ = 0.184, F(7,90) = 2.89, *p* < 0.009). However, similar to findings with added sugar, there was not a significant association of percent calories from glucose on the MD of the right cingulum, prefrontal connections (ß = 2.25 × 10^−6^, sr = 0.15, *p* < 0.13). Likewise, similar to added sugar, the overall model for glucose associations with MD of the right cingulum, prefrontal connections was driven by significant associations with decreased age and increased family income. These result are different from the effects we observed in the volume of the right CA2/3 because MD was only associated with fructose intake and not added sugar or glucose intake.

### 3.4. Post-hocs for Diffusion Imaging Measures

Because MD is defined as the average diffusivity, it is not as biologically specific as axial diffusivity (AD) or radial diffusivity (RD). Therefore, we ran post-hoc analyses using Model 4 only to determine whether the observed association of increased fructose intake on MD of the right cingulum, prefrontal connections, was driven by axial diffusivity (AD), radial diffusivity (RD), or both. The AD for the right cingulum, prefrontal connections was significantly associated with increased percentage of calories from fructose (ß = 4.62 × 10^−6^, sr = 0.27, *p* < 0.005; Model 4). The RD for the right cingulum, prefrontal connections was significantly associated with increased percentage of calories from fructose (ß = 2.89 × 10^−6^, sr = 0.23, *p* < 0.01; Model 4). This pattern was quite similar to the pattern observed with MD. Comparing the effect sizes of sr = 0.27 for AD and sr = 0.23 for RD, we conclude that the effects were similar, but slightly larger for AD.

## 4. Discussion

In this study, we examined the influence of dietary sugar intake on hippocampal neuroanatomy, both gray matter and white matter, in children. We found that increased volume in the right hippocampal CA2/3 subfield was associated with increased consumption of the monosaccharides, fructose or glucose, and/or added sugars in general, while increased MD in the right cingulate-prefrontal cortex connections were only associated with increased dietary intake of fructose. We found associations on the right but not the left hemisphere, which is broadly consistent with prior work showing lateralities on the impact of environmental insults in the hippocampus [58,59]. These associations between dietary sugar intake and hippocampal volume and fructose intake and cingulate-prefrontal cortex connections remained significant after adjusting for child’s intracranial volume, age, sex, BMI, SES, and prenatal exposures suggesting an effect of dietary fructose, added sugar, and glucose intake on hippocampal volume and an effect of dietary fructose on cingulate-prefrontal cortex white matter connectivity that is independent of a number of potential confounding factors.

The observed association between dietary sugar intake and increased hippocampal volume during childhood may be driven by a few factors. A larger hippocampal CA2/3 volume during childhood could be due to a delay in synaptic pruning, a process that typically occurs during early adolescence. During adolescence, the hippocampus reaches peak volume, and begins to undergo synaptic pruning, eliminating unused connections [60,61], and prior studies have shown that the CA3 hippocampal subfield gradually begins to decrease in volume during mid childhood [61,62]. Work by Vuong et al. found that synaptic pruning is delayed in juvenile rats exposed in utero to obese mothers with GDM [63]. In their findings, inflammation and recruitment of microglial cells occurred during post-natal development in the hippocampus, along with a reduction in synaptic pruning, and the animals presented with altered hippocampal morphology. These findings suggest that prenatal environmental insults can result in hippocampal inflammation, reductions in synaptic pruning and altered hippocampal development [63]. Interestingly, Hershey et al., found that children with Type 1 diabetes had increased hippocampal volume, despite the commonly reported decreased volume in adults with Type 1 and Type 2 diabetes [64]. There is substantial research that a diet high in added sugar contributes to a pro-inflammatory environment [13,19,65]. Therefore, increased volume in the CA2/3 hippocampal subfield may be due to inflammation and/or a delay in synaptic pruning. Future studies that examine inflammation specifically, such as T2 scans for gliosis, could potentially address this possibility [66].

Notably, we found that the CA2/3 hippocampal subfield was preferentially impacted by dietary sugar intake. Coincidentally, the CA3 subfield is among the last subfields to undergo postnatal maturation, paralleling DG development [67,68]. Additionally, substantial work in animals has shown that the CA3 subfield is altered by a host of prenatal and early life environmental insults, such as prenatal exposure to GDM [69,70] or postnatal exposure to chronic stress [71,72,73]. Therefore, our findings are in line with prior research in animals indicating a preferential sensitivity of the CA3 subfield. It is worth noting, due to the predetermined boundaries delineated by FreeSurfer, we were unable to decipher the boundary between the CA2 and CA3 subfield. Future studies that incorporate manual tracing should be considered to confirm if excessive added sugar intake preferentially impacts the CA3 subfield of the hippocampus.

Many studies have observed that MD decreases while FA increases over the course of development [52,53,54,55]. We found that increased dietary fructose intake was associated with increased mean diffusivity (MD) in the white matter cingulum tract that connects the right hippocampus and the right prefrontal cortex. MD has been shown to increase in many different white matter diseases that lead to demyelination, dysmyelination, and/or wallerian degeneration [74,75,76]. For example, increases in MD have been observed prior to the appearance of lesions on a gadolinium-enhanced scan in multiple sclerosis, which is a complex disease that involves not only demyelination and wallerian degeneration, but also edema and inflammation, indicating that changes in the MD may be sensitive to pre-lesion changes in the blood brain barrier [77]. Patients with chronic epilepsy and hippocampal sclerosis show increased MD values in the hippocampus [78,79]. Together, these results are consistent with the idea that the increased MD values associated with fructose could reflect an inflammatory process, possibly associated with a loss of myelin, or delayed axonal pruning.

Because mean diffusivity is a weighted average of axial (AD) and radial diffusivity (RD), we conducted post-hoc analyses on these measures to obtain more biological specificity about what might be underlying the observed associations with MD. In general, AD is thought to reflect intracellular water mobility and is influenced by the integrity and arrangement of axonal membranes and cytoskeletal proteins, while RD is thought to reflect more of the extracellular water mobility and is primarily influenced by myelin [80]. Previous studies have shown that over the course of normal neurodevelopment, both RD and AD decrease with age [81,82,83,84,85]. Decreases in RD are typically attributed to myelination, while the decreases in AD are thought to correspond more to axonal pruning [86]. In our study, we found that both AD and RD were increased in the cingulum tract in children with increased dietary fructose intake, consistent with the idea that increases in fructose intake could be associated with a decrease or delay in the myelination process and the normal pruning process. Future studies could use multi-compartment diffusion imaging models, such as Neurite Orientation Dispersion and Density Imaging (NODDI), to further investigate this tract to determine whether these changes in diffusivity are more related to cellular density and/or orientation dispersion, thus adding more biological specificity [87].

Self-reported dietary assessments have known limitations, including under-reporting of dietary intake. Given that eating behavior can vary from day to day, the collection of multiple 24-hr recalls on non-consecutive days is recommended to improve the accuracy of habitual dietary intake estimates [88]. Some studies have recommended obtaining at least three 24-hr dietary recalls on non-consecutive days to provide better accuracy of energy intake [36]. Dietary assessments in our study were obtained from repeated 24-hr recalls obtained during in-person visits on non-consecutive days. We obtained two days of dietary assessments on the majority of participants, and four days of dietary assessments on a subset of 35 participants. We implemented techniques to improve the accuracy of our dietary data collection method, including the Multiple Pass 24-h recall method, which was previously shown to significantly reduce levels of under-reporting [89]. We also used a consensus recall method, in which the child and parent were interviewed together, which has been suggested to improve the accuracy of dietary assessments compared to interviews with the child or parent alone [90]. Estimates of total energy intake in our cohort are similar to national averages for children in this age range [91], while the average percent calories from added sugar in our cohort was 2.5% lower than the average of 16.4% reported in the 2009 to 2012 National Health and Nutrition Examination Survey for children age 6–11 [92]. Thus, while total energy intake estimates in our cohort are in line with those of national averages of children in the U.S., it is possible that our cohort under-reported intake of foods and beverages that contain added sugars. It is also possible that the children in our cohort consumed, on average, slightly lower amounts of added sugars than average children in the U.S. population.

In summary, our findings suggest that increases in dietary fructose are associated with alterations in hippocampal structure and connectivity in children. These findings should be interpreted cautiously given the limitations of self-reported dietary intake assessments, and it is important to note that our observations are correlational and do not confer causality. Future studies that include experimental designs that manipulate dietary intake of fructose and/or added sugars are necessary to determine the effects of fructose and added sugar on hippocampal structure and connectivity during childhood. Moreover, the potential cognitive consequences of the observed associations between dietary fructose and alterations in hippocampal structure and connectivity remains an important consideration. Our findings support the need for future studies that include cognitive testing in addition to neuroimaging to examine whether increased dietary fructose intake in childhood is associated with altered hippocampal structure and hippocampal function in childhood.

## Figures and Tables

**Table 1:** Child demographic and clinical characteristics

	Mean (SD) or N (%) ^1^	Range
Age, years	8.55 (1.03)	7.33–11.34
BMI, kg/m^2^	19.00 (4.12)	13.62–34.01
BMI percentile	69.96 (27.33)	5.28–99.58
BMI z-score	0.77 (1.08)	−1.78–2.64
BMI category	Healthy-weight: 61 (59%) Overweight: 16 (16%) Obese: 26 (25%)	
Sex	Boys: 41 (40%) Girls: 62 (60%)	
Tanner Stage of Pubertal Development	Tanner stage 1: 94 (91%) Tanner stage 2: 5 (5%) Tanner stage 3: 3 (3%) Tanner stage 4: 1 (1%)	
Energy Intake (kcal)	1763 (359)	825–2708
Percent Calories from Added Sugar (%)	13.91 (6.89)	2.65–39.90
Percent Calories from Glucose (%)	4.32 (1.81)	1.21–8.40
Percent Calories from Fructose (%)	4.57 (2.19)	0.93–11.44

^1^ Percentages were rounded to the nearest percent, therefore sum of variables do not equal 100%; BMI: body mass index.

**Table 2:** Familial demographics.

	Mean (SD) or N (%) ^1^
Maternal education ^2^	LN: 21 (20%) SC: 29 (28%) CN: 51 (50%)
Mother’s race/ethnicity	Hispanic: 59 (57%) Black: 11 (11%) Non-Hispanic White: 20 (19%) Other: 13 (13%)
Family income ^2^	0–$30 K: 10 (10%) $30 K–50 K: 29 (29%) $50 K–70 K: 33 (33%) $70 K–90 K: 14 (14%) ≥$90 K: 15 (15%)

^1^ Percentages were rounded to the nearest percent, therefore sum of variables do not equal 100%; ^2^ Missing maternal education and family income data from 2 participants.

**Table 3:** Associations between dietary fructose intake and the volume of right CA2/3.

Predictor Variables	Model 1 ß (sr)	Model 2 ß (sr)	Model 3 ß (sr)	Model 4 ß (sr)	Model 5 ß (sr)
Percent Calories from Fructose	3.34 (0.25) **	2.56 (0.19) *	2.80 (0.21) *	2.70 (0.19) *	3.33 (0.24) **
Age, years		−1.06 (−0.04)	−1.14 (−0.04)	−1.27 (−0.04)	−1.71 (−0.06)
Sex (1, male; 0, female)		5.17 (0.07)	5.44 (0.08)	6.18 (0.08)	2.52 (0.03)
Intracranial Volume (mm^3^)		1.31 × 10^−4^ (0.49) ***	1.30 × 10^−4^ (0.48) ***	1.34 × 10^−4^ (0.47) ***	1.24 × 10^−4^ (0.43) ***
BMI z-score			3.13 (0.11)	3.05 (0.11)	5.80 (0.20) *
Family income (1: <$30 K; 5: >$90 K)				−1.67 (−0.01)	−1.90 (−0.07)
Mom’s education (CN)					
LN				3.24 (0.04)	2.56 (0.03)
SC				2.01 (0.03)	1.81 (0.03)
Maternal GDM (1, yes; 0, no)					4.29 (0.07)
Maternal pre-pregnancy BMI, kg/m^2^					−1.31 (−0.28) ***
					
R^2^	0.064	0.365	0.378	0.380	0.459
∆R^2^		0.301	0.013	0.002	0.079
∆F		16.78 ***	2.13	0.11	6.56 **

Model 1 is percent calories from fructose only; Model 2 includes age, sex and intracranial volume; Model 3 includes BMI z-score; Model 4 includes family income and mom’s education; Model 5 includes prenatal exposures to maternal GDM and maternal pre-pregnancy BMI. Successive models include all covariates from earlier models. BMI: body mass index; LN: <high school; SC: some college; CN: college and post-graduate; GDM: gestational diabetes mellitus; sr = semi-partial r; * *p* < 0.05; ** *p* < 0.01; *** *p* < 0.001.

**Table 4:** Effects of fructose consumption on the mean diffusivity (MD) of right cingulum, prefrontal connections.

Predictor Variables	Model 1 ß (sr)	Model 2 ß (sr)	Model 3 ß (sr)	Model 4 ß (sr)	Model 5 ß (sr)
Percent Calories from Fructose	2.51 × 10^−6^ (0.21) *	2.66 × 10^−6^ (0.22) *	2.79 × 10^−6^ (0.23) *	3.51 × 10^−6^ (0.28) **	3.64 × 10^−6^ (0.29) **
Age, years		−7.52 × 10^−6^ (−0.28) **	−7.50 × 10^−6^ (−0.28) **	−6.78 × 10^−6^ (−0.25) **	−6.95 × 10^−6^ (−0.26) **
Sex (1, male; 0, female)		−9.79 × 10^−6^ (−0.19)	-9.82 × 10^−6^ (−0.19)	−8.65 × 10^−6^ (−0.17)	−8.37 × 10^−6^ (−0.16)
BMI z-score			1.67 × 10^−6^ (0.07)	1.68 × 10^−6^ (0.07)	2.40 × 10^−6^ (0.10)
Family income (1: <$30 K; 5: >$90 K)				6.71 × 10^−6^ (0.29) **	6.56 × 10^−6^ (0.28) **
Mom’s education (CN)					
LN				3.64 × 10^−6^ (0.05)	5.09 × 10^−6^ (0.07)
SC				4.69 × 10^−6^ (0.08)	5.67 × 10^−6^ (0.09)
Maternal GDM (1, yes; 0, no)					−3.99 × 10^−6^ (−0.08)
Maternal pre-pregnancy BMI, kg/m^2^					−3.51 × 10^−7^ (−0.09)
					
R^2^	0.044	0.152	0.157	0.243	0.257
∆R^2^		0.108	0.005	0.086	0.014
∆F		6.87 **	0.57	3.42 *	0.83

Model 1 includes percent calories from fructose only; Model 2 includes age and sex; Model 3 includes BMI z-score; Model 4 includes family income and mom’s education; Model 5 includes maternal GDM and maternal pre-pregnancy BMI. Successive models include all covariates from earlier models. BMI: body mass index; LN: <high school; SC: some college; CN: college and post-graduate; GDM: gestational diabetes mellitus; sr = semi-partial r; * *p* < 0.05; ** *p* < 0.01.

## Supplementary Materials

The following are available online at https://www.mdpi.com/2072-6643/12/4/909/s1, Figure S1: Raw data plots of added sugar consumption and the mean diffusivity of the right cingulum, prefrontal connections, Figure S2: Raw data plots of added sugar consumption and the volume of the right CA2/3 subfield of the hippocampus, Figure S3: Raw data plots of glucose consumption and the mean diffusivity of the right cingulum, prefrontal connections, Figure S4: Raw data plots of glucose consumption and the volume of the right CA2/3 subfield of the hippocampus, Table S1: Table of hippocampal and total brain volumes.

## References

[1] Bray G.A., Nielsen S.J., Popkin B.M. (2004). Consumption of high-fructose corn syrup in beverages may play a role in the epidemic of obesity. Am. J. Clin. Nutr..

[2] Basciano H., Federico L., Adeli K. (2005). Fructose, insulin resistance, and metabolic dyslipidemia. Nutr. Metab..

[3] Tappy L., Le K.A. (2010). Metabolic Effects of Fructose and the Worldwide Increase in Obesity. Physiol. Rev..

[4] Johnson R.J., Segal M.S., Sautin Y., Nakagawa T., Feig D.I., Kang D.-H., Gersch M.S., Benner S., Sánchez-Lozada L.G. (2007). Potential role of sugar (fructose) in the epidemic of hypertension, obesity and the metabolic syndrome, diabetes, kidney disease, and cardiovascular disease. Am. J. Clin. Nutr..

[5] Lindqvist A., Baelemans A., Erlanson-Albertsson C. (2008). Effects of sucrose, glucose and fructose on peripheral and central appetite signals. Regul. Pept..

[6] Page K.A., Chan O., Arora J., Belfort-DeAguiar R., Dzuira J., Roehmholdt B., Cline G.W., Naik S., Sinha R., Constable R.T. (2013). Effects of Fructose vs Glucose on Regional Cerebral Blood Flow in Brain Regions Involved with Appetite and Reward Pathways. JAMA.

[7] Luo S., Monterosso J.R., Sarpelleh K., Page K.A. (2015). Differential effects of fructose versus glucose on brain and appetitive responses to food cues and decisions for food rewards. Proc. Natl. Acad. Sci. USA.

[8] Teff K.L., Elliott S.S., Tschöp M., Kieffer T.J., Rader D., Heiman M., Townsend R.R., Keim N.L., D’Alessio D., Havel P.J. (2004). Dietary Fructose Reduces Circulating Insulin and Leptin, Attenuates Postprandial Suppression of Ghrelin, and Increases Triglycerides in Women. J. Clin. Endocrinol. Metab..

[9] Cha S.H., Wolfgang M., Tokutake Y., Chohnan S., Lane M.D. (2008). Differential effects of central fructose and glucose on hypothalamic malonyl—CoA and food intake. Proc. Natl. Acad. Sci. USA.

[10] Erlanson-Albertsson C., Lindqvist A. (2010). Fructose affects enzymes involved in the synthesis and degradation of hypothalamic endocannabinoids. Regul. Pept..

[11] Lowette K., Roosen L., Tack J., Vanden Berghe P. (2015). Effects of high-fructose diets on central appetite signaling and cognitive function. Front. Nutr..

[12] Lakhan S.E., Kirchgessner A. (2013). The emerging role of dietary fructose in obesity and cognitive decline. Nutr. J..

[13] Hsu T.M., Kanoski S.E. (2014). Blood-brain barrier disruption: Mechanistic links between Western diet consumption and dementia. Front. Aging Neurosci..

[14] Davidson T.L., Chan K., Jarrard L.E., Kanoski S.E., Clegg D.J., Benoit S.C. (2009). Contributions of the Hippocampus and Medial Prefrontal Cortex to Energy and Body Weight Regulation. Hippocampus.

[15] Hannapel R.C., Henderson Y.H., Nalloor R., Vazdarjanova A., Parent M.B. (2017). Ventral hippocampal neurons inhibit postprandial energy intake. Hippocampus.

[16] Agrawal R., Gomez-Pinilla F. (2012). “Metabolic syndrome” in the brain: Deficiency in omega-3 fatty acid exacerbates dysfunctions in insulin receptor signalling and cognition. J. Physiol..

[17] Wu H.-W., Ren L.-F., Zhou X., Han D.-W. (2015). A high-fructose diet induces hippocampal insulin resistance and exacerbates memory deficits in male Sprague-Dawley rats. Nutr. Neurosci..

[18] Hsu T.M., Konanur V.R., Taing L., Usui R., Kayser B.D., Goran M.I., Kanoski S.E. (2015). Effects of sucrose and high fructose corn syrup consumption on spatial memory function and hippocampal neuroinflammation in adolescent rats. Hippocampus.

[19] Djordjevic A., Bursać B., Veličković N., Vasiljević A., Matić G. (2015). The impact of different fructose loads on insulin sensitivity, inflammation, and PSA-NCAM-mediated plasticity in the hippocampus of fructose-fed male rats. Nutr. Neurosci..

[20] Van der Borght K., Köhnke R., Göransson N., Deierborg T., Brundin P., Erlanson-Albertsson C., Lindqvist A. (2011). Reduced neurogenesis in the rat hippocampus following high fructose consumption. Regul. Pept..

[21] Ross A.P., Bartness T.J., Mielke J.G., Parent M.B. (2009). A high fructose diet impairs spatial memory in male rats. Neurobiol. Learn. Mem..

[22] Hebben N., Corkin S., Eichenbaum H., Shedlack K. (1985). Diminished ability to interpret and report internal states after bilateral medial temporal resection: Case, H.M. Behav. Neurosci..

[23] Rozin P., Dow S., Moscovitch M., Rajaram S. (1998). What Causes Humans to Begin and End a Meal? A Role for Memory for What Has Been Eaten, as Evidenced by a Study of Multiple Meal Eating in Amnesic Patients. Psychol. Sci..

[24] Maller J.J., Welton T., Middione M., Callaghan F.M., Rosenfeld J.V., Grieve S.M. (2019). Revealing the Hippocampal Connectome through Super-Resolution 1150-Direction Diffusion MRI. Sci. Rep..

[25] Chudasama Y., Doobay V.M., Liu Y. (2012). Hippocampal-Prefrontal Cortical Circuit Mediates Inhibitory Response Control in the Rat. J. Neurosci..

[26] Hsu T.M., Noble E.E., Liu C.M., Cortella A.M., Konanur V.R., Suarez A.N., Reiner D.J., Hahn J.D., Hayes M.R., Kanoski S.E. (2018). A hippocampus to prefrontal cortex neural pathway inhibits food motivation through glucagon-like peptide-1 signaling. Mol. Psychiatry.

[27] Sweeney P., Yang Y. (2015). An excitatory ventral hippocampus to lateral septum circuit that suppresses feeding. Nat. Commun..

[28] Russo C., Russo A., Pellitteri R., Stanzani S. (2017). Hippocampal Ghrelin-positive neurons directly project to arcuate hypothalamic and medial amygdaloid nuclei. Could they modulate food-intake?. Neurosci. Lett..

[29] Geha P., Cecchi G., Constable R.T., Abdallah C., Small D.M. (2017). Reorganization of brain connectivity in obesity. Hum. Brain Mapp..

[30] Alexander A.L., Lee J.E., Lazar M., Field A.S. (2007). Diffusion Tensor Imaging of the Brain. Neurotherapeutics.

[31] Koebnick C., Langer-Gould A.M., Gould M.K., Chao C.R., Iyer R.L., Smith N., Chen W., Jacobsen S.J. (2012). Sociodemographic Characteristics of Members of a Large, Integrated Health Care System: Comparison with US Census Bureau Data. Perm. J..

[32] Defining Childhood Obesity|Overweight & Obesity|CDC. https://www.cdc.gov/obesity/childhood/defining.html.

[33] Marshall W.A., Tanner J.M. (1969). Variations in pattern of pubertal changes in girls. Arch. Dis. Child..

[34] Marshall W.A., Tanner J.M. (1970). Variations in the pattern of pubertal changes in boys. Arch. Dis. Child..

[35] Rasmussen A.R., Wohlfahrt-Veje C., Tefre de Renzy-Martin K., Hagen C.P., Tinggaard J., Mouritsen A., Mieritz M.G., Main K.M. (2015). Validity of self-assessment of pubertal maturation. Pediatrics.

[36] Johnson R.K., Driscoll P., Goran M.I. (1996). Comparison of Multiple-Pass 24-Hour Recall Estimates of Energy Intake with Total Energy Expenditure Determined by the Doubly Labeled Water Method in Young Children. J. Am. Diet. Assoc..

[37] Schakel S.F., Buzzard I.M., Gebhardt S.E. (1997). Procedures for Estimating Nutrient Values for Food Composition Databases. J. Food Compos. Anal..

[38] Iglesias J.E., Augustinack J.C., Nguyen K., Player C.M., Player A., Wright M., Roy N., Frosch M.P., McKee A.C., Wald L.L. (2015). A computational atlas of the hippocampal formation using ex vivo, ultra-high resolution MRI: Application to adaptive segmentation of in vivo MRI. NeuroImage.

[39] Backhausen L.L., Herting M.M., Buse J., Roessner V., Smolka M.N., Vetter N.C. (2016). Quality Control of Structural MRI Images Applied Using FreeSurfer—A Hands-On Workflow to Rate Motion Artifacts. Front. Neurosci..

[40] Calvo-Ochoa E., Hernández-Ortega K., Ferrera P., Morimoto S., Arias C. (2014). Short-term high-fat-and-fructose feeding produces insulin signaling alterations accompanied by neurite and synaptic reduction and astroglial activation in the rat hippocampus. J. Cereb. Blood Flow Metab..

[41] Molteni R., Barnard R.J., Ying Z., Roberts C.K., Gómez-Pinilla F. (2002). A high-fat, refined sugar diet reduces hippocampal brain-derived neurotrophic factor, neuronal plasticity, and learning. Neuroscience.

[42] Al-Amin M., Zinchenko A., Geyer T. (2018). Hippocampal subfield volume changes in subtypes of attention deficit hyperactivity disorder. Brain Res..

[43] Tamnes C.K., Walhovd K.B., Engvig A., Grydeland H., Krogsrud S.K., Østby Y., Holland D., Dale A.M., Fjell A.M. (2014). Regional Hippocampal Volumes and Development Predict Learning and Memory. Dev. Neurosci..

[44] Jenkinson M., Beckmann C.F., Behrens T.E.J., Woolrich M.W., Smith S.M. (2012). FSL. Neuroimage.

[45] Cabeen R.P., Laidlaw D.H., Toga A.W. Quantitative Imaging Toolkit: Software for Interactive 3D Visualization, Processing, and Analysis of Neuroimaging Datasets. Proceedings of the Annual Meeting of the International Society for Magnetic Resonance in Medicine (ISMRM).

[46] Arsigny V., Fillard P., Pennec X., Ayache N. (2006). Log-Euclidean metrics for fast and simple calculus on diffusion tensors. Magn. Reson. Med..

[47] Zhang H., Yushkevich P.A., Alexander D.C., Gee J.C. (2006). Deformable registration of diffusion tensor MR images with explicit orientation optimization. Med. Image Anal..

[48] Zhang S., Peng H., Dawe R.J., Arfanakis K. (2011). Enhanced ICBM Diffusion Tensor Template of the Human Brain. Neuroimage.

[49] Cabeen R.P., Bastin M.E., Laidlaw D.H. (2016). Kernel Regression Estimation of Fiber Orientation Mixtures in Diffusion MRI. Neuroimage.

[50] Wakana S., Caprihan A., Panzenboeck M.M., Fallon J.H., Perry M., Gollub R.L., Hua K., Zhang J., Jiang H., Dubey P. (2007). Reproducibility of quantitative tractography methods applied to cerebral white matter. Neuroimage.

[51] Catani M., Thiebaut de Schotten M. (2008). A diffusion tensor imaging tractography atlas for virtual in vivo dissections. Cortex.

[52] Neil J., Miller J., Mukherjee P., Hüppi P.S. (2002). Diffusion tensor imaging of normal and injured developing human brain—A technical review. NMR Biomed..

[53] Snook L., Paulson L.-A., Roy D., Phillips L., Beaulieu C. (2005). Diffusion tensor imaging of neurodevelopment in children and young adults. NeuroImage.

[54] Mukherjee P., McKinstry R.C. (2006). Diffusion tensor imaging and tractography of human brain development. Neuroimaging Clin. N. Am..

[55] Bonekamp D., Nagae L.M., Degaonkar M., Matson M., Abdalla W.M., Barker P.B., Mori S., Horská A. (2007). Diffusion tensor imaging in children and adolescents: Reproducibility, hemispheric, and age-related differences. Neuroimage.

[56] Drewnowski A., Rehm C.D. (2013). Energy intakes of US children and adults by food purchase location and by specific food source. Nutr. J..

[57] CDC Know Your Limit for Added Sugars. https://www.cdc.gov/nutrition/data-statistics/know-your-limit-for-added-sugars.html.

[58] Zach P., Mrzílková J., Stuchlík A., Valeš K. (2010). Delayed Effects of Elevated Corticosterone Level on Volume of Hippocampal Formation in Laboratory Rat. Physiol. Res..

[59] Teicher M.H., Anderson C.M., Polcari A. (2012). Childhood maltreatment is associated with reduced volume in the hippocampal subfields CA3, dentate gyrus, and subiculum. Proc. Natl. Acad. Sci. USA.

[60] Andersson J.L., Skare S., Ashburner J. (2003). How to correct susceptibility distortions in spin-echo echo-planar images: Application to diffusion tensor imaging. Neuroimage.

[61] Tamnes C.K., Bos M.G.N., van de Kamp F.C., Peters S., Crone E.A. (2018). Longitudinal development of hippocampal subregions from childhood to adulthood. Dev. Cogn. Neurosci..

[62] Canada K.L., Ngo C.T., Newcombe N.S., Geng F., Riggins T. (2019). It’s All in the Details: Relations Between Young Children’s Developing Pattern Separation Abilities and Hippocampal Subfield Volumes. Cereb. Cortex.

[63] Vuong B., Odero G., Rozbacher S., Stevenson M., Kereliuk S.M., Pereira T.J., Dolinsky V.W., Kauppinen T.M. (2017). Exposure to gestational diabetes mellitus induces neuroinflammation, derangement of hippocampal neurons, and cognitive changes in rat offspring. J. Neuroinflamm..

[64] Hershey T., Perantie D.C., Wu J., Weaver P.M., Black K.J., White N.H. (2010). Hippocampal Volumes in Youth with Type 1 Diabetes. Diabetes.

[65] Beilharz J.E., Maniam J., Morris M.J. (2016). Short-term exposure to a diet high in fat and sugar, or liquid sugar, selectively impairs hippocampal-dependent memory, with differential impacts on inflammation. Behav. Brain Res..

[66] Lee J.K., Ekstrom A.D., Ghetti S. (2014). Volume of hippocampal subfields and episodic memory in childhood and adolescence. NeuroImage.

[67] Jabès A., Lavenex P.B., Amaral D.G., Lavenex P. (2011). Postnatal Development of the Hippocampal Formation: A Stereological Study in Macaque Monkeys. J. Comp. Neurol..

[68] Lu D., He L., Xiang W., Ai W.-M., Cao Y., Wang X.S., Pan A., Luo X.-G., Li Z., Yan X.-X. (2013). Somal and Dendritic Development of Human CA3 Pyramidal Neurons from Midgestation to Middle Childhood: A Quantitative Golgi Study. Anat. Rec..

[69] Golalipour M.J., Kafshgiri S.K., Ghafari S. (2012). Gestational diabetes induced neuronal loss in CA1 and CA3 subfields of rat hippocampus in early postnatal life. Folia Morphol..

[70] Lotfi N., Hami J., Hosseini M., Haghir D., Haghir H. (2016). Diabetes during pregnancy enhanced neuronal death in the hippocampus of rat offspring. Int. J. Dev. Neurosci..

[71] Schoenfeld T.J., McCausland H.C., Morris H.D., Padmanaban V., Cameron H.A. (2017). Stress and Loss of Adult Neurogenesis Differentially Reduce Hippocampal Volume. Biol. Psychiatry.

[72] Kumar R.S., Narayanan S.N., Kumar N., Nayak S. (2018). Exposure to Enriched Environment Restores Altered Passive Avoidance Learning and Ameliorates Hippocampal Injury in Male Albino Wistar Rats Subjected to Chronic Restraint Stress. Int. J. Appl. Basic Med. Res..

[73] Baran S.E., Campbell A.M., Kleen J.K., Foltz C.H., Wright R.L., Diamond D.M., Conrad C.D. (2005). Combination of high fat diet and chronic stress retracts hippocampal dendrites. Neuroreport.

[74] Guo A.C., Jewells V.L., Provenzale J.M. (2001). Analysis of normal-appearing white matter in multiple sclerosis: Comparison of diffusion tensor MR imaging and magnetization transfer imaging. AJNR Am. J. Neuroradiol..

[75] Pierpaoli C., Barnett A., Pajevic S., Chen R., Penix L.R., Virta A., Basser P. (2001). Water diffusion changes in Wallerian degeneration and their dependence on white matter architecture. Neuroimage.

[76] Horsfield M.A., Jones D.K. (2002). Applications of diffusion-weighted and diffusion tensor MRI to white matter diseases—A review. NMR Biomed..

[77] Werring D.J., Brassat D., Droogan A.G., Clark C.A., Symms M.R., Barker G.J., MacManus D.G., Thompson A.J., Miller D.H. (2000). The pathogenesis of lesions and normal-appearing white matter changes in multiple sclerosis: A serial diffusion MRI study. Brain.

[78] Wieshmann U.C., lark C.A.C., Symms M.R., Barker G.J., Birnie K.D., Shorvon S.D. (1999). Water diffusion in the human hippocampus in epilepsy. Magn. Reson. Imaging.

[79] Yoo S.Y., Chang K.-H., Song I.C., Han M.H., Kwon B.J., Lee S.H., Yu I.K., Chun C.-K. (2002). Apparent diffusion coefficient value of the hippocampus in patients with hippocampal sclerosis and in healthy volunteers. AJNR Am. J. Neuroradiol..

[80] Schwartz E.D., Cooper E.T., Fan Y., Jawad A.F., Chin C.-L., Nissanov J., Hackney D.B. (2005). MRI diffusion coefficients in spinal cord correlate with axon morphometry. Neuroreport.

[81] Zhang Y., Zhang J., Oishi K., Faria A.V., Jiang H., Li X., Akhter K., Rosa-Neto P., Pike G.B., Evans A. (2010). Atlas-guided tract reconstruction for automated and comprehensive examination of the white matter anatomy. Neuroimage.

[82] Lebel C., Walker L., Leemans A., Phillips L., Beaulieu C. (2008). Microstructural maturation of the human brain from childhood to adulthood. NeuroImage.

[83] Bava S., Thayer R., Jacobus J., Ward M., Jernigan T.L., Tapert S.F. (2010). Longitudinal characterization of white matter maturation during adolescence. Brain Res..

[84] Suzuki Y., Matsuzawa H., Kwee I.L., Nakada T. (2003). Absolute eigenvalue diffusion tensor analysis for human brain maturation. NMR Biomed..

[85] Kumar R., Nguyen H.D., Macey P.M., Woo M.A., Harper R.M. (2012). Regional brain axial and radial diffusivity changes during development. J. Neurosci. Res..

[86] Bockhorst K.H., Narayana P.A., Liu R., Ahobila-Vijjula P., Ramu J., Kamel M., Wosik J., Bockhorst T., Hahn K., Hasan K.M. (2008). Early postnatal development of rat brain: In vivo diffusion tensor imaging. J. Neurosci. Res..

[87] Zhang H., Schneider T., Wheeler-Kingshott C.A., Alexander D.C. (2012). NODDI: Practical in vivo neurite orientation dispersion and density imaging of the human brain. NeuroImage.

[88] Tucker K.L. (2007). Assessment of usual dietary intake in population studies of gene—Diet interaction. Nutr. Metab. Cardiovasc. Dis..

[89] Moshfegh A.J., Rhodes D.G., Baer D.J., Murayi T., Clemens J.C., Rumpler W.V., Paul D.R., Sebastian R.S., Kuczynski K.J., Ingwersen L.A. (2008). The US Department of Agriculture Automated Multiple-Pass Method reduces bias in the collection of energy intakes. Am. J. Clin. Nutr..

[90] Foster E., Bradley J. (2018). Methodological considerations and future insights for 24-hour dietary recall assessment in children. Nutr. Res..

[91] Herrick K.A., Rossen L., Parsons R., Dodd K. (2018). Estimating usual dietary intake from National Health and Nutrition Examination Survey data using the National Cancer Institute method. Natl. Cent. Health Stat..

[92] Vos M.B., Kaar J.L., Welsh J.A., Van Horn L.V., Feig D.I., Anderson C.A.M., Patel M.J., Munos J.C., Krebs N.F., Xanthakos S.A. (2017). Added Sugars and Cardiovascular Disease Risk in Children. Circulation.

