# Supplementary material for: Dietary Fructose Intake and Hippocampal Structure and Connectivity during Childhood
Source: Nutrients. 2020 Mar 26;12(4):909. doi: 10.3390/nu12040909 (PMC7230400; doi:10.3390/nu12040909)
Supplement: Supplementary file 1 [file nutrients-12-00909-s001.zip › SupplementalData/Nutrients_SupplementalData.docx]

**Supplemental Data**

**S1. Table of hippocampal and total brain volumes**

| **Brain Volume (n=104)** | **Mean (SD)** | **Range** |
| --- | --- | --- |
| Intracranial Volume (mm^3^) | 1414025.23 (133227.31) | 1,140,715~1,870,666 |
| Left Hippocampal Volume (mm^3^) | 3439.58 (378.95) | 2,676.99~ 5,513.39 |
| Right Hippocampal Volume (mm^3^) | 3480.83 (324.67) | 2,797.96~ 4,342.92 |
| Left CA1 Volume (mm^3^) | 639.21 (84.05) | 437.85~1,024.09 |
| Right CA1 Volume (mm^3^) | 653.90 (77.46) | 484.89~856.60 |
| Left CA3 Volume (mm^3^) | 202.34 (30.31) | 133.63~ 306.80 |
| Right CA3 Volume (mm^3^) | 219.51 (28.99) | 150.41~303.35 |
| Left CA4 Volume (mm^3^) | 255.48 (29.73) | 197.74~400.34 |
| Right CA4 Volume (mm^3^) | 264.24 (28.45) | 203.35~339.48 |
| Left Dentate Gyrus Volume (mm^3^) | 301.80 (34.42) | 234.25~464.16 |
| Right Dentate Gyrus Volume (mm^3^) | 309.90 (32.99) | 240.26~ 398.11 |
| Left Subiculum Volume (mm^3^) | 435.39 (48.44) | 354.77~677.71 |
| Right Subiculum Volume (mm^3^) | 424.72 (42.16) | 325.98~531.22 |

**Supplemental Figure Legends**

**Figure S1. Associations between added sugar consumption and the mean diffusivity of the right cingulum, prefrontal connections.** Graphs show the unadjusted model with no covariates. Solid line indicates the best fit linear trend, with dotted lines showing the confidence interval. Red points indicate subjects with greater than 10% of calories from added sugar.

**Figure S2. Associations between added sugar consumption and the volume of the right CA2/3 subfield of the hippocampus.** Graphs show the unadjusted model with no covariates. Solid line indicates the best fit linear trend, with dotted lines showing the confidence interval. Red points indicate subjects with greater than 10% of calories from added sugar.

**Figure S3. Associations between glucose consumption and the mean diffusivity of the right cingulum, prefrontal connections.** Graphs show the unadjusted model with no covariates. Solid line indicates the best fit linear trend, with dotted lines showing the confidence interval.

**Figure S4. Associations between glucose consumption and the volume of the right CA2/3 subfield of the hippocampus.** Graphs show the unadjusted model with no covariates. Solid line indicates the best fit linear trend, with dotted lines showing the confidence interval.
