# Supplementary figures and images for: Dietary Fructose Intake and Hippocampal Structure and Connectivity during Childhood
Source: Nutrients. 2020 Mar 26;12(4):909. doi: 10.3390/nu12040909 (PMC7230400; doi:10.3390/nu12040909)

## Unadjusted Model

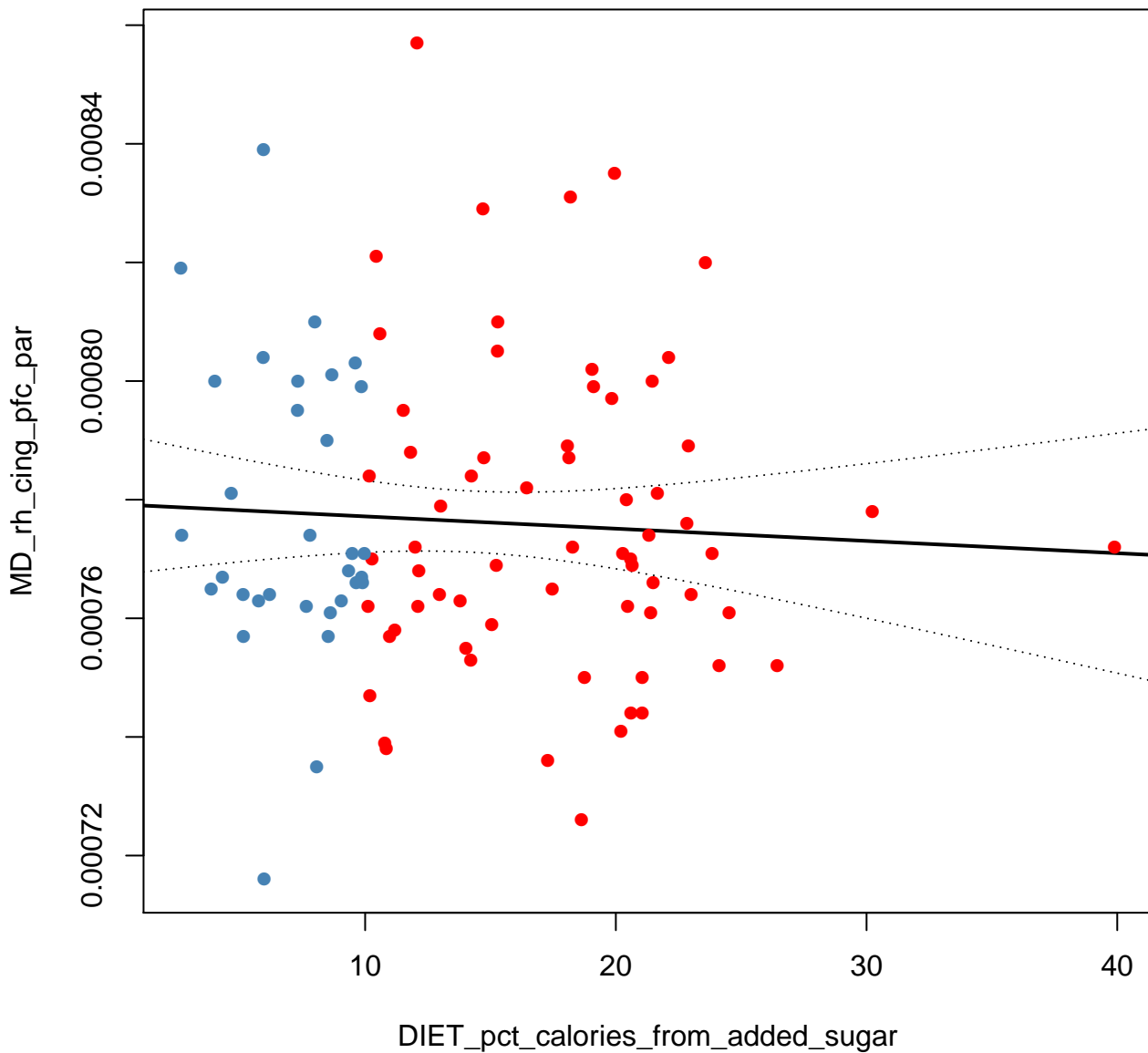

Supplement: Supplementary file 1 [file nutrients-12-00909-s001.zip › SupplementalData/Nutrients_FigureS1.pdf]

## Unadjusted Model

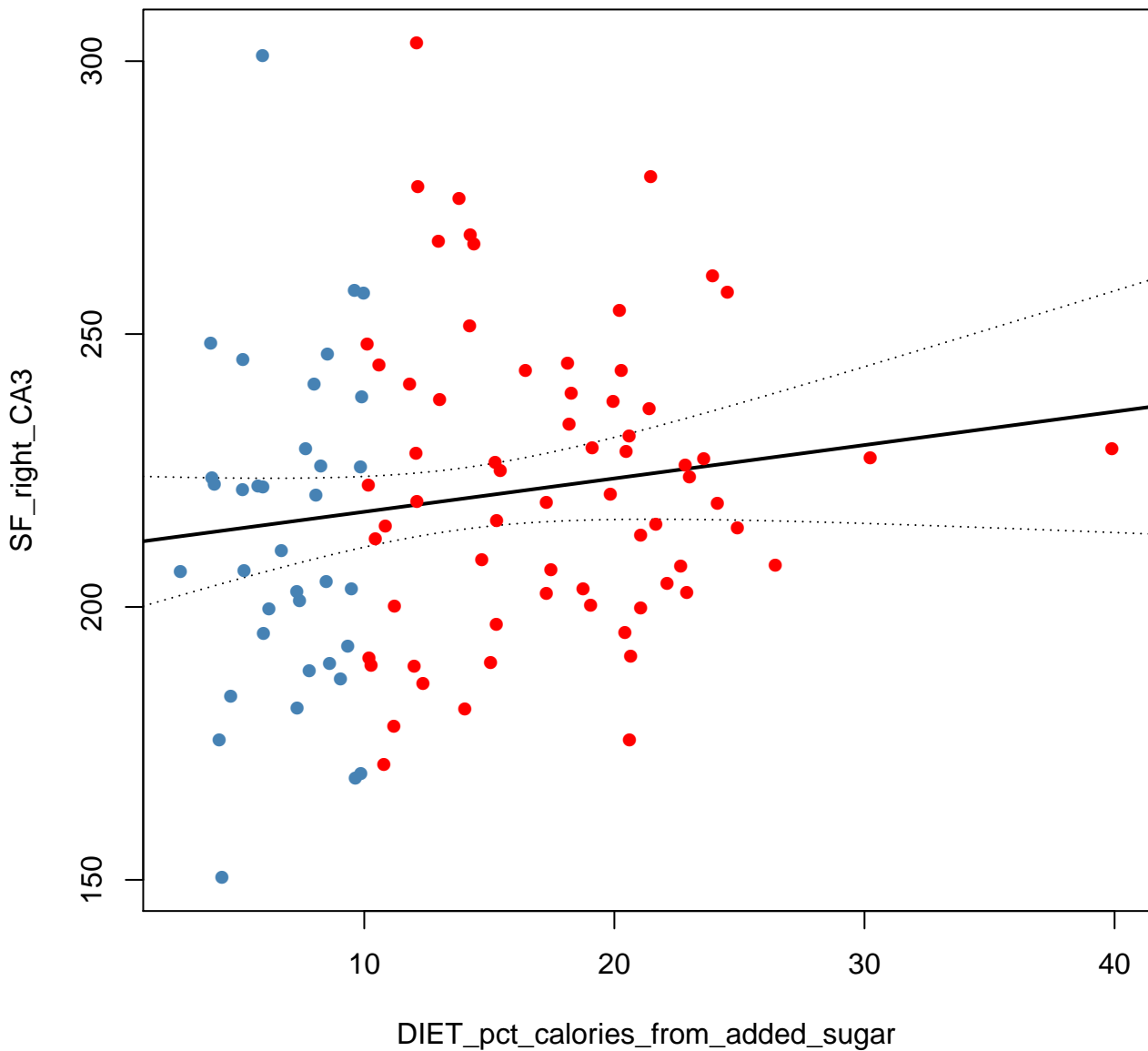

Supplement: Supplementary file 1 [file nutrients-12-00909-s001.zip › SupplementalData/Nutrients_FigureS2.pdf]

## Unadjusted Model

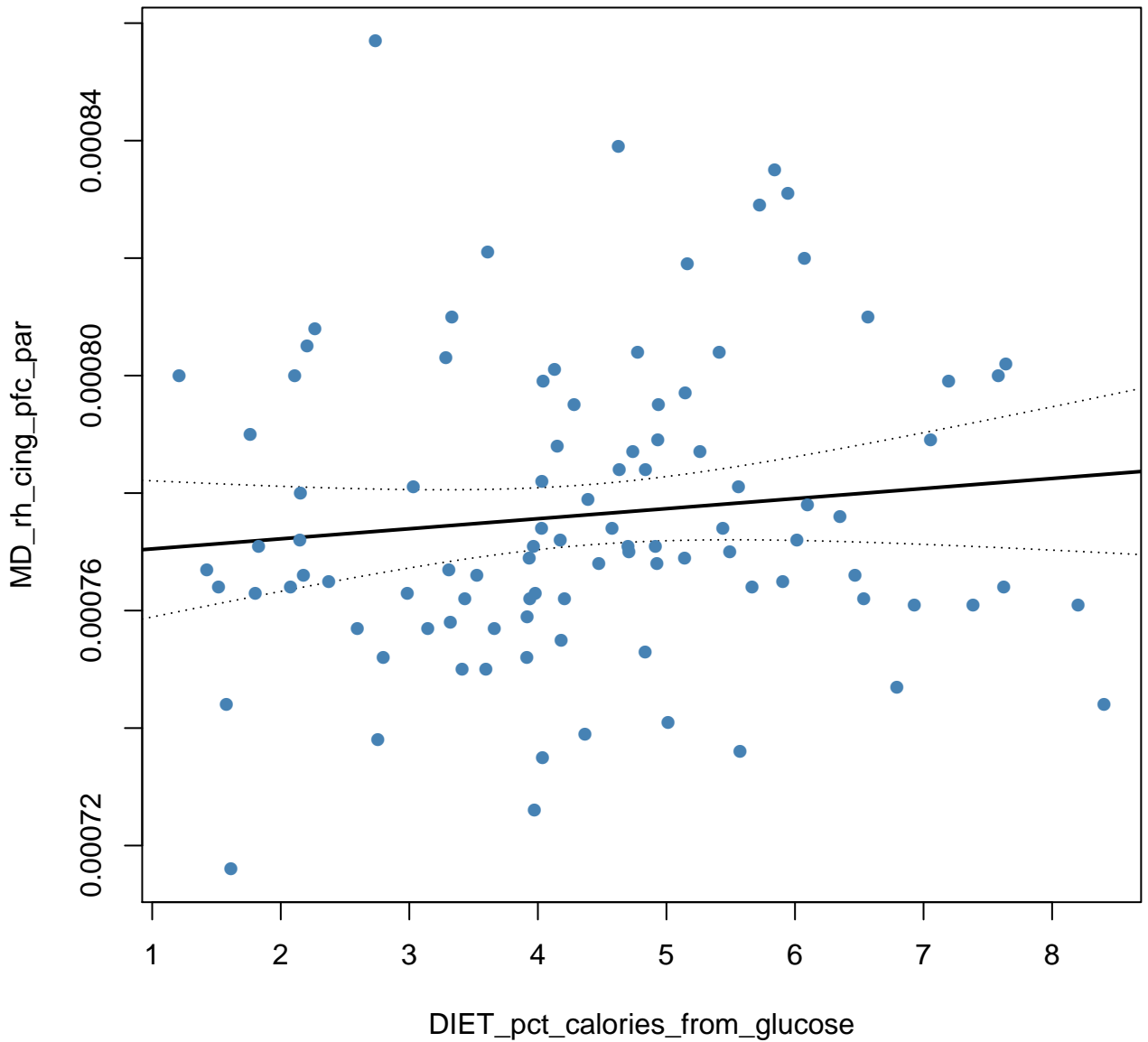

Supplement: Supplementary file 1 [file nutrients-12-00909-s001.zip › SupplementalData/Nutrients_FigureS3.pdf]

## Unadjusted Model

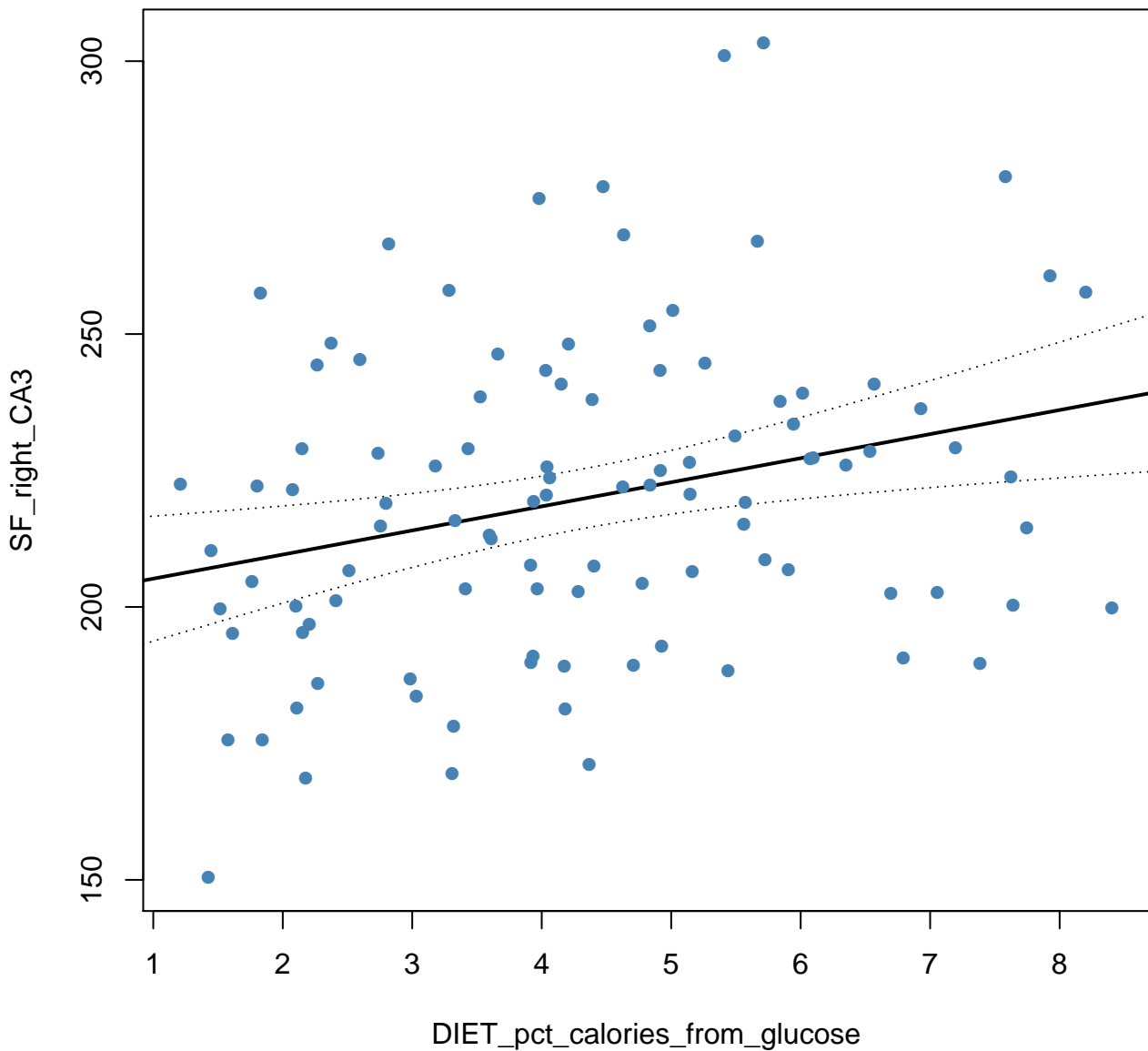

Supplement: Supplementary file 1 [file nutrients-12-00909-s001.zip › SupplementalData/Nutrients_FigureS4.pdf]
